# Rebuttal to “Comment on: Fate After the Mustard Procedure for d-Transposition of the Great Arteries: Impact of Age, Complexity, and Atrial Tachyarrhythmias: A Single Center Experience” by Iman Akram

**DOI:** 10.1007/s00246-024-03593-8

**Published:** 2024-07-22

**Authors:** Ulrich Krause

**Affiliations:** Department of Pediatric Cardiology, Intensive Care Medicine and Neonatology, University Medical Center, Georg-August-University Göttingen, Robert-Koch-Str. 40, 37099 Göttingen, Germany

To the editor:

We would like to take the opportunity to respond to the letter by Dr. Iman Akram on our previous manuscript entitled “Fate After the Mustard Procedure for d-Transposition of the Great Arteries: Impact of Age, Complexity, and Atrial Tachyarrhythmias: A Single Center Experience” [1]. Though there is a small number of multicenter studies on the fate of patients with d-transposition of the great arteries (d-TGA) after the atrial switch procedure, our knowledge on the natural course of those individuals is still sparse. In our manuscript, we admit, that one of the limitations of our study is its single center character and we also have recognized the study by Broberg et al. [2]. We do not agree with the view, that potential selection bias impacts the results of our study as all patients with d-TGA who had a Mustard procedure at our institution between 1969 and 1994 were enrolled. We did not include patients after the arterial switch operation, as comparison of long-term outcome after the atrial switch procedure versus the arterial switch procedure in patients with d-TGA was explicitly not aim of our study. Likewise, our study did not aim to evaluate the impact of general cardiovascular risk factors on the fate of individuals after the atrial switch procedure for d-TGA. Therefore, we focused on analysis of specific risk factors associated with morbidity and mortality in patients with d-TGA after the atrial switch procedure. Finally, we acknowledge the importance of in-depth analysis of quality of life (QOL) of patients with d-TGA after the atrial switch procedure. A retrospective, longitudinal study without the use of specific questionnaires, however, might not suffice to obtain reliable results on QOL in this group of patients.

## Data Availability

No datasets were generated or analysed during the current study.
